# C-755-03. Improving Efficiency of Outpatient Pediatric Burn Sedation—a Process Improvement Study

**DOI:** 10.1093/jbcr/irag033.039

**Published:** 2026-04-06

**Authors:** Caden Bizer, Nicole Baier, Emiko Rivera, Clifford C Sheckter

**Affiliations:** Santa Clara Valley Medical Center, San Jose, CA; Santa Clara Valley Medical Center, San Jose, CA; Santa Clara Valley Medical Center, San Jose, CA; Stanford University, San Jose, CA

## Abstract

**Introduction:**

Children with burns often require sedation for wound debridement and dressing changes. When safe and feasible, these patients can be managed outpatient—avoiding admission. Our burn center has increasing growth of this treatment paradigm leading to system strain and limiting additional sedation sessions. We aimed to improve the efficiency of our outpatient pediatric sedation program to increase capacity.

**Methods:**

A Plan-Do-Study-Act cycle was initiated with nurses, surgeons, pediatricians, and pharmacists to reduce sedation duration in a verified burn center’s pediatric sedation unit. The Plan stage had the primary goal of decreasing sedation time during dressing changes without compromising quality. A needs survey identified contributors to sedation prolongation including waiting on wound supplies housed in the Burn Center (pediatric sedation occurs on the pediatric ward) and waiting on prescription topicals housed in central or Burn Center pharmacies. A unique pediatric sedation cart was created containing burn dressings, wound care instructions, and comfort items (stuffies). Pharmacists stocked topicals (bacitracin, silver sulfadiazine, antimicrobial foam, etc) in the pediatric pharmacy. The Do stage included nursing and pharmacy management ensuring the cart and Pyxis were stocked. The duration of sedation dressing was measured in minutes before and after implementation, and times were compared using interrupted time series regression. Additionally, a paired t-test evaluated differences in sedation/pain medications before and after implementation. The Act phase included adjusting the number of sedation sessions based on gains in time.

**Results:**

Prior to implementation, dressing changes were routinely exceeding 35 minutes for an average burn size of 6% total body surface area. After implementation, interrupted time series demonstrated a 15.0-minute reduction in dressing time (*p*=.013). Analysis of ketamine use showed no statistically significant difference pre- vs. post-implementation (2.9 mg/kg vs 3.3 mg/kg, *p*=.40). Similarly, oxycodone (2.3 mg vs 2.4 mg, *p*=.67) and midazolam (7.4 mg vs 8.1 mg), *p*=.58) doses did not change. The decrease in time per sedation session has since been accompanied by an annual increase in pediatric sedation procedures from 106 to 157 (51 more procedures per year, 48% increase in capacity).

**Conclusions:**

A Plan-Do-Study-Act process improvement initiative successfully reduced the duration of outpatient pediatric sedation procedures. Standardizing supply carts and stocking topical medications on unit were drivers of success. Increased capacity has allowed for additional sedation procedures.

**Applicability of Research to Practice:**

Nursing driven process improvement projects can improve burn center capacity to care for pediatric burns as outpatients. Other centers could consider adopting similar strategies.

**Funding for the Study:**

N/A.

